# Fully Differential Touch Screen Controller with Wide Input Dynamic Range for Thin Displays

**DOI:** 10.3390/s20030837

**Published:** 2020-02-04

**Authors:** Chang-Ju Lee, Jong Kang Park, Han-Eol Seo, Junho Huh, Jung-Hoon Chun

**Affiliations:** 1 College of Information and Communication Engineering, Sungkyunkwan University, Suwon 16419, Korea; 2 Samsung Electronics, Hwaseong 18448, Korea

**Keywords:** touch screen panel, display noise, fully differential charge amplifier, wide input dynamic range, thin display

## Abstract

As today’s smartphone displays become thinner, the coupling capacitance between the display electrodes and touch screen panel (TSP) electrodes is increasing significantly. The increased capacitance easily introduces time-varying display signals into the TSP, deteriorating the touch performance. In this research, we demonstrate that the maximum peak display noise in the time domain is approximately 30% of the maximum voltage difference of the display grayscale through analysis of the structure and operation of displays. Then, to mitigate display noise, we propose a circuit solution that uses a fully differential charge amplifier with an input dynamic range wider than the maximum peak of the display noise. A test chip was fabricated using a 0.35 μm CMOS process and achieved a signal-to-noise ratio of 41 dB for a 6-mm-diameter metal pillar touch when display pulses with 5-V swing were driven at 100 kHz.

## 1. Introduction

Today’s smartphone displays are becoming slimmer due to the form factor of these devices. In these slim display modules, the display and touch screen panel (TSP) are placed extremely close to each other, thereby increasing the coupling capacitance between the electrodes of the two panels. The increased capacitance makes it easier for the display drive signals to interfere with the sensing signals of the TSP, which can significantly degrade touch performance.

To address the display noise problem, a number of circuit techniques have been proposed [1,2,3,4,5,6]. One such technique is to subtract the measured noise in a display common plate from the input signals of the touch-sensing ICs [3]. However, because this method uses the average noise measured in the full display area, it does not reflect local differences in noise depending on the position of the sensor channels. Another method is to separate the touch-sensing period from the display driving period [7]. Since this method does not generate any display noise during the touch-sensing period, the signal-to-noise ratio (SNR) against the display noise is theoretically infinite. However, in high-resolution displays, the display quality may worsen due to the insufficient writing time of a display image. In addition, it can also reduce the touch-sensing time, which may seriously degrade immunity against noise sources other than the display noise. Increasing the driving voltage for TSPs can also be a useful solution, but it requires a fabrication process that forms transistors with high breakdown voltages and consumes higher power to charge and discharge parasitic capacitance around the sensor electrodes [8].

In other methods, analog front-end (AFE) circuits using differential sensing schemes have shown excellent noise immunity against display noise by exploiting the fact that display noise coupled to adjacent channels has almost the same magnitude and phase [2,3,4,5,6,9,10,11,12]. Because they calculate touch coordinates by using the difference between the signals sensed in two adjacent channels, the display noise can be significantly reduced. The differential sensing methods for touch sensing have been implemented in various forms to date. The first scheme used the output difference between two single-ended charge amplifiers (SE-CAs), but each output is easily saturated to the supply or ground by strong display noise in spite of the simple implementation [9,10,12]. In a more advanced circuit, pseudo-differential amplifiers in which one input of the amplifier is fixed to a reference voltage were used, but unfortunately, this approach still suffers from the same saturation problem [2]. To prevent the charge amplifier output from saturating, several techniques that apply fully differential charge amplifiers (FD-CAs) at the first stage of an AFE were proposed [6,11]. Among them, two studies, [6,11], showed particularly excellent display noise immunity, both of which implemented input common-mode feedback (ICMFB) circuits as well as FD-CAs. The ICMFB circuits strongly force the average voltage of two inputs of the FD-CA to a specific reference voltage, typically half the supply voltage, so that the input voltages do not obviate from the input dynamic range of the amplifier. In this scheme, since the auxiliary ICMFB circuit, not the main amplifier, supplies current to the TSP sensors in response to display noise, the FD-CA outputs can avoid saturation. Nevertheless, because of the small differential feedback gain of the ICMFB circuit, the differential components in the noise generated from the ICMFB circuit can distort touch signals in the FD-CA outputs, resulting in a reduced SNR. This problem will be discussed in detail in Section 3.

The work presented in this paper also implemented a fully differential amplifier at the first stage of AFE circuits. However, instead of using an ICMFB circuit, by analyzing the magnitude of the CA input terminal voltage fluctuating in response to display noise, we designed an FD-CA with a wide input dynamic range that can cope with the worst display noise. The AFE circuit with this amplifier has two distinct advantages compared with the previous work. First, there is no SNR decrease due to the differential noise of the ICMFB circuit. Second, even in ultrathin touchscreen panels with considerably large coupling capacitance with display electrodes, the AFE can be implemented as a compact and low-power circuit. In ultrathin panels, the ICMFB circuits should have a large current supply capability to charge the large coupling capacitance, resulting in an increase in the power consumption and area of the circuit. However, if the introduced display noise voltage does not exceed the input dynamic range of the amplifier, since the proposed circuit responds to the difference in input capacitances, not the capacitance itself, the performance requirements of the AFE amplifier can be relaxed.

Section 2 analyzes the temporal characteristics of the display noise introduced from the display to the TSP. Then, Section 3 describes the details of circuit implementation and Section 4 evaluates the experimental results and performance of the implemented test chip.

## 2. Transient Characteristics of Display Noise 

**Figure 1:**
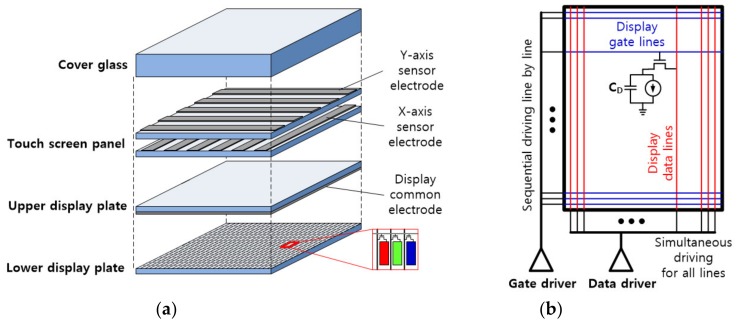
Composition of display panel with touch screen panel: (**a**) Vertical structure of display panel; (**b**) Display driving circuits and operation in the lower display plate of (**a**).

Although there have been many previous studies on display noise, most of them focused on developing noise-immune circuits. A quantitative analysis of display noise itself has not attracted much attention. In this section, we analyzed the transient characteristics of display noise by establishing an electric circuit model representing displays with a TSP. First, it is necessary to understand the structure of display modules embedded with a TSP and their operation. The TSP is usually located on top of a display panel to increase touch sensitivity, as shown in Figure 1a, and the common electrode (or cathode electrode) of the display is located on the upper side of the display pixel circuits [1,4]. 

Figure 1b illustrates a display module with numerous data lines and gate lines to write image data to the display pixels. Here, the data lines transfer voltages corresponding to grayscale data to target pixels, and the gate lines turn thin-film transistors (TFTs) on or off to connect a storage capacitor in the pixel to the corresponding data line. Though there are some exceptions [13], in general, high-resolution displays write an image by the matrix-addressing method used in memory chips [14]. In such displays, the gate lines are sequentially driven line by line, whereas all of the data lines are driven simultaneously; therefore, most of the display noise results from driving the data lines.

**Figure 2:**
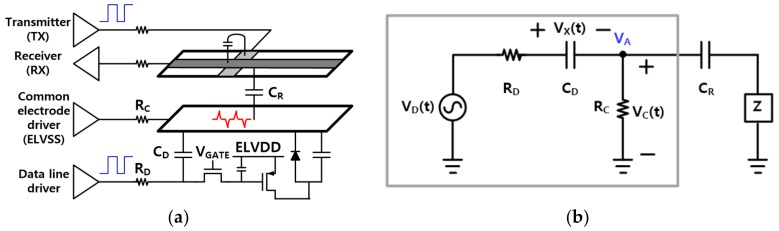
(**a**) Simplified circuit configuration of the OLED displays embedded with a TSP and (**b**) the equivalent electric circuit model for a display noise analysis.

To date, organic light-emitting diode (OLED) displays have been the most popular type of displays in smartphones. Figure 2a shows the simplified vertical structure of the OLED display, which includes display pixels, display driving circuitry, and parasitic components for display noise analysis. In most low-power OLED displays, the pixel current variation is less than 100 uA for a horizontal write period, and the common electrode resistance is about several ohms. Hence, the potential change of the common electrode is only a few millivolts at most, which is significantly smaller than the potential variation caused by coupling the data lines (this coupling effect will be discussed further in the remainder of this section). Thus, the pixel current of OLED displays can be ignored in the electric model. On the other hand, because each pixel changes its internal voltage only when the corresponding gate line is turned on, the display noise coupled through the storage capacitance of the display is also very small compared to the noise coupled with the data lines. In conclusion, the mechanism of generating display noise can be described in such a manner that the voltage driving of data lines for a write operation is transferred to the common electrode through the coupling capacitance between the electrodes.

Based on the above considerations, the displays can be modeled as the simple equivalent electric circuit shown in Figure 2b. The write operation of the data lines is modeled with the display noise source V_D_(t). R_D_ is the equivalent parallel resistance of the routing resistors of all of the data lines, C_D_ is the coupling capacitance between all of the data lines and the display common electrode, and R_C_ is the resistance from the display common electrode driver to the common electrode. The coupling capacitance between a sensing electrode and the display common electrode is denoted by C_R_. Finally, for simplicity of the circuit analysis, the readout circuits of AFEs can be modeled as an impedance of Z ranging from zero to infinity.

The maximum voltage of node V_A_ can be calculated from the circuit in the gray box of Figure 2 b, assuming that an impedance magnitude of C_R_ is sufficiently greater than R_C_. This assumption is valid for most mobile OLED displays. The current density of pixels required to represent the maximum grayscale is approximately 0.1 nA/um^2^ [15], and several hundred microamperes of current may flow through the common electrode in a 6-inch display with FHD resolution (1920 x 1080 pixels). Hence, R_C_ is usually managed as less than 10 Ω to prevent non-uniform display images due to IR (current x resistance) drop in the common electrode. Meanwhile, in recent years, the thickness of encapsulation layer in flexible OLED displays is challenging to even less than 10 um for long-term bending reliability [16], and the decrease in thickness leads to a significant increase of C_R_. Excessively large C_R_ limits the operating frequencies of the touch sensing signal by lowering the upper cut-off frequency of the touchscreen [10,17], so many commercial displays use mesh-shaped metal electrodes to ensure that its capacitance does not exceed 1 nF per channel. In addition, since most touch sensors operate at frequencies of 1 MHz or less, the calculated maximum V_A_ voltage can be regarded as the maximum peak of the display noise in the time domain. 

Equations (1) and (2) are differential equations describing the gray box circuit. V_D_(t) can be expressed as a step pulse with a slope to emulate a write operation of data lines so that it consists of two periods, a slewing period and a settling period, as shown in Equation (3). Here, the slewing time, t_1_, is determined by the slew rate of the source amplifiers in the display driver ICs, and the height of the pulse, A, can have a maximum value corresponding to the difference between the two grayscale voltages for black and white.

(1) $$\frac{dV_{X}\left(t\right)}{dt}+\frac{1}{\left(R_{D}+R_{C}\right)C_{D}}V_{X}\left(t\right)=\frac{1}{\left(R_{D}+R_{C}\right)C_{D}}V_{D}\left(t\right)$$

(2) $$V_{C}\left(t\right)=\frac{R_{C}}{R_{D}+R_{C}}\left(V_{D}\left(t\right)-V_{X}\left(t\right)\right)$$

(3) $$V_{D}\left(t\right)=\left\{\begin{array}{l} \frac{A}{t_{1}}\cdot t\;\;\;,\;0\leq t<t_{1}\\ A\;\;\;\;\;,\;t_{1}\leq t \end{array}\right.$$

Solution:

$\mathrm{Let}\;\tau =\left(R_{D}+R_{C}\right)C_{D},$

i) $0\leq t\leq t_{1}$
  (4) $$V_{C}\left(t\right)=\frac{R_{C}}{R_{D}+R_{C}}\cdot \frac{\tau }{t_{1}}\cdot A\left(1-e^{-\frac{t}{\tau }}\right)$$
ii) $t_{1}\leq t$
  (5) $$V_{C}\left(t\right)=\frac{R_{C}}{R_{D}+R_{C}}\cdot \frac{\tau }{t_{1}}\cdot A\left(1-e^{-\frac{t_{1}}{\tau }}\right)\cdot e^{-\frac{\left(t-t_{1}\right)}{\tau }}$$
iii) $\mathrm{the}\;\mathrm{maximum}\;\mathrm{of}\;V_{C}\left(t\right)\;\mathrm{at}\;t=t_{1}$
  (6) $$V_{C,\mathrm{MAX}}=\frac{R_{C}}{R_{D}+R_{C}}\cdot \frac{\tau }{t_{1}}\cdot A\left(1-e^{-\frac{t_{1}}{\tau }}\right)$$

**Figure 3:**
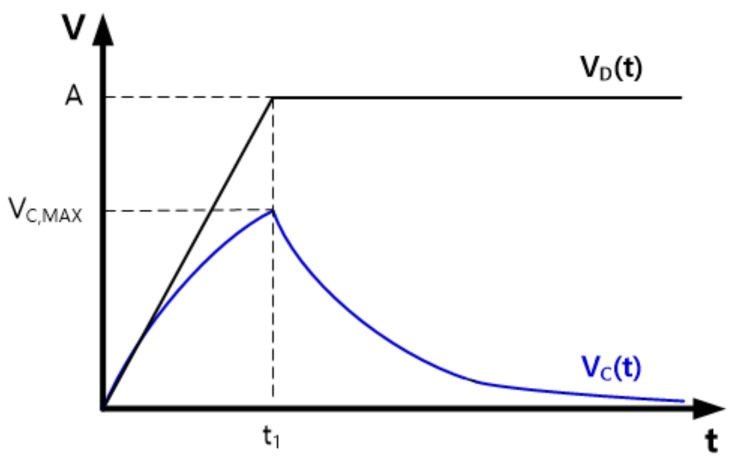
Solution of differential equation for V_C_(t).

The mathematical solution of V_C_(t) is summarized from (4) to (6), and its graphical shape is shown in Figure 3. The solution of (6) means that display noise increases as t_1_ decreases or when R_D_ is much smaller than R_C_. However, reducing t_1_ below τ increases the power consumption of the display driver ICs and does not effectively improve the write speed of the display because the passive network of the data lines determines the slope of the input pulse written to the pixels. Conversely, if t_1_ is longer than τ, the display pixels may not be sufficiently charged within a horizontal writing period, and therefore the image quality of the display may deteriorate. Hence, normal displays set t_1_ to a value very close to τ. On the other hand, in OLED displays, R_C_ typically has a smaller value than R_D_ because a large R_C_ makes the write time of the display longer, resulting in an insufficient settlement of the image data in the display pixels. Moreover, a large R_C_ value may cause a non-uniform display image over the entire display area due to a large IR drop, as discussed before. Therefore, R_C_/(R_C_ + R_D_) is expected to have a value of about 0.5 or less in most panels.

Applying the conditions considered above for slewing time and resistance values to Equation (6), we can conclude that the temporal maximum value of the display noise is less than 30% of the maximum voltage change of the display driving pulse.

## 3. Design of Touch Screen Controller

### 3.1. Chip Configuration

**Figure 4:**
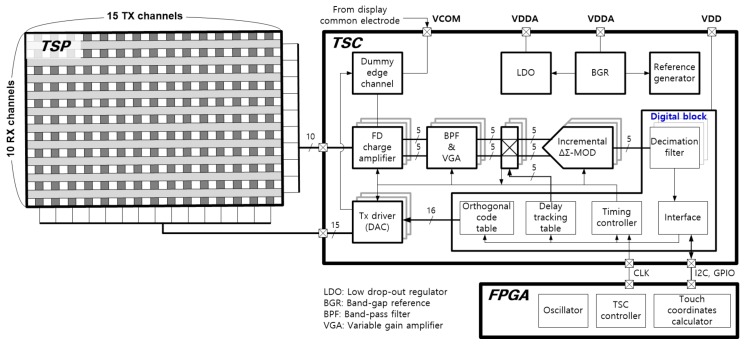
Touch screen panel sensor system including the proposed TSC.

Figure 4 illustrates the overall touch sensing system for this study. The TSP has 15 TX channels and 10 RX channels, and the touch screen controller (TSC) includes a plurality of drivers, sensing circuits and control blocks for mutual capacitance sensing. The TSC converts a number of sensing signals changed by touch into a stream of digital data and sends it to the touch coordinates calculator in the field programmable gate array (FPGA) chip. Finally, the FPGA chip calculates the touch coordinates with additional noise filtering and controls the timing of the entire system.

**Figure 5:**
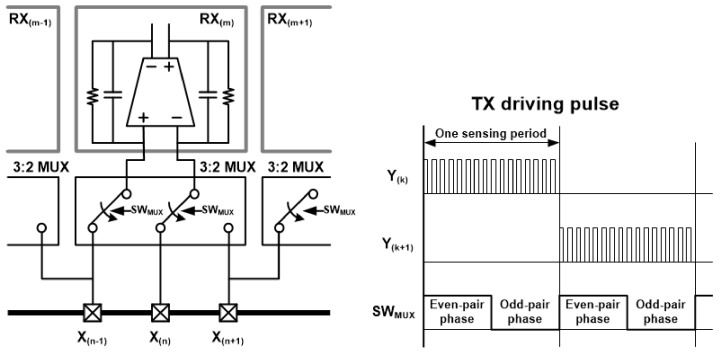
A 3:2 channel multiplexing configuration for proposed fully differential charge amplifiers and timing chart.

The first stage of TSC is designed with FD-CAs to reduce the display noise commonly introduced into two adjacent RX sensing channels, and it amplifies only the difference between the touch signals passing through two mutual capacitances. To reduce the chip area, the number of readout circuits is implemented as half of the total number of RX channels. To obtain the capacitance differences in all pairs of adjacent RX channels, the connection configuration of the differential sensing pairs is alternated over two frames using a 3-to-2 multiplexer, as shown in Figure 5.

The noise components included in the output of the FD-CA are strongly attenuated by a second-order band-pass filter (BPF) and the output signal is properly amplified by a variable gain amplifier (VGA). In the demodulation block, while a square wave with the same frequency as the TX driving signal and a duty rate of 50% is multiplied to the touch signal, the frequency of the touch signal is moved down to the DC. Since the delay amounts of the touch signal through different nodes of the TSP may be different from each other, we matched the phases of the signal and the demodulation square wave node by node to maximize the demodulated touch signal power [6,7]. The signal processed in the analog domain is modulated into a bit stream using a delta-sigma modulator for an oversampling ADC and converted it into a digital code proportional to the magnitude of the touch signal by a second-order *Col.* filter [18]. 

### 3.2. Fully Differential Charge Amplifier

**Figure 6:**
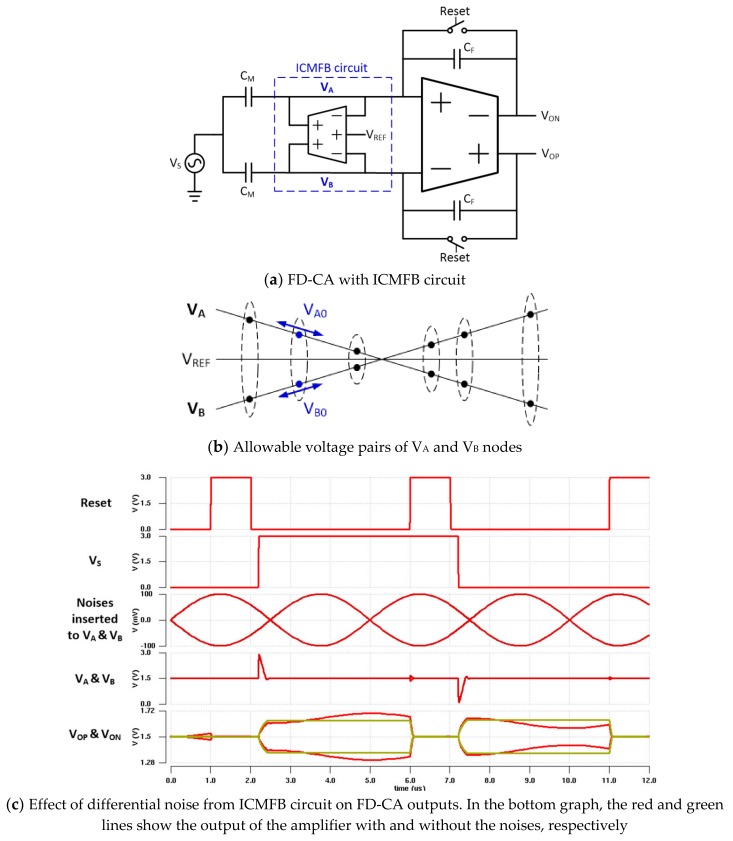
FD-CA with ICMFB and the output characteristics by differential noises of the ICMFB circuit.

As mentioned before, the previous studies [6,11] implemented fully differential sensing circuits with ICMFB function. The ICMFB circuit biases the average voltage of two amplifier inputs to a specific voltage, such as V_REF_ in Figure 6a, and ensures that the average voltage does not obviate from the input dynamic range of the amplifier. This operation is very effective for reducing display noise because the display noise couples with almost the same phase and magnitude to adjacent channels.

Two inputs of the FD-CA with the ICMFB circuit can have a vertical pair of voltages on the V_A_ and V_B_ lines in Figure 6b. Although the input pair V_A_(t) and V_B_(t) should be determined by the difference in mutual capacitances and the closed-loop gain of the FD-CA in the ideal operation, the pairs can be changed from the initial values for various reasons. For example, if differential noise is generated at the output of the ICMFB amplifier when the initial voltages of V_A_ and V_B_ nodes are given as V_A0_ and V_B0_, respectively, which are determined by the difference in mutual capacitances, charges of different polarities are supplied to the two inputs of the FD-CA. In this case, since the average voltage of the two lines does not change, the negative feedback operation, in which each of the input voltages of the FD-CA is returned to the initial value, does not work at all. Instead, as the two inputs move in opposite directions, the differential output of the FD-CA varies. This means that the output of the FD-CA is very sensitive to the differential noise of the ICMFB output. Figure 6c shows the simulation result that monitors the outputs of the FD-CA when two sine waves with 100-mV amplitudes and 180-degree phase difference are applied to the V_A_ and V_B_ nodes. The simulation result confirms that the differential noise of the ICMFB circuit can distort the received touch signal.

**Figure 7:**
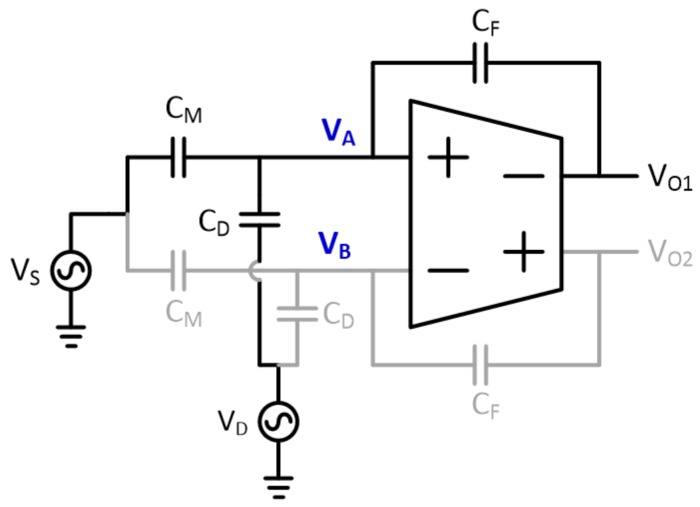
Capacitive coupling model of signal and display noise to calculate the input voltage of FD-CA.

To solve this problem, instead of using an ICMFB circuit, we designed a fully differential amplifier with a wide input dynamic voltage range. As shown in Figure 7, the FD-CA input voltages are determined by three capacitances, C_M_, C_D_, C_F_, and their respective driving voltages. If C_D_ is overwhelmingly larger than the other capacitances, as is true for ultrathin displays, the input voltages of the FD-CA are determined by the display noise coupled by C_D_ as derived in Equation (7).

(7) $$\Delta V_{A}\cdot \left(C_{D}+C_{M}+C_{F}\right)=\Delta V_{N}\cdot C_{D}+\Delta V_{S}\cdot C_{M}+\Delta V_{O}\cdot C_{F}\;\Delta V_{A}=\Delta V_{N}\cdot \frac{C_{D}}{C_{D}+C_{M}+C_{F}}+\Delta V_{S}\cdot \frac{C_{M}}{C_{D}+C_{M}+C_{F}}+\Delta V_{O}\cdot \frac{C_{F}}{C_{D}+C_{M}+C_{F}}\approx \Delta V_{N}\;\left(\mathrm{if}\;C_{D}\gg C_{M},C_{F}\right)$$

In Section 2, we found that the maximum peak value of the display noise is less than 30% of the maximum voltage change of the display inputs. In commercial OLED displays for mobile devices, the maximum voltage change of grayscale is typically less than 5 V [19]; therefore, the worst display noise would have a peak voltage below 1.5 V. Therefore, even under the worst noise condition, a fully differential amplifier that has a supply voltage of more than 3 V and a rail-to-rail input dynamic range can perform the differential sensing operation without signal distortion regardless of the C_D_ value. 

**Figure 8:**
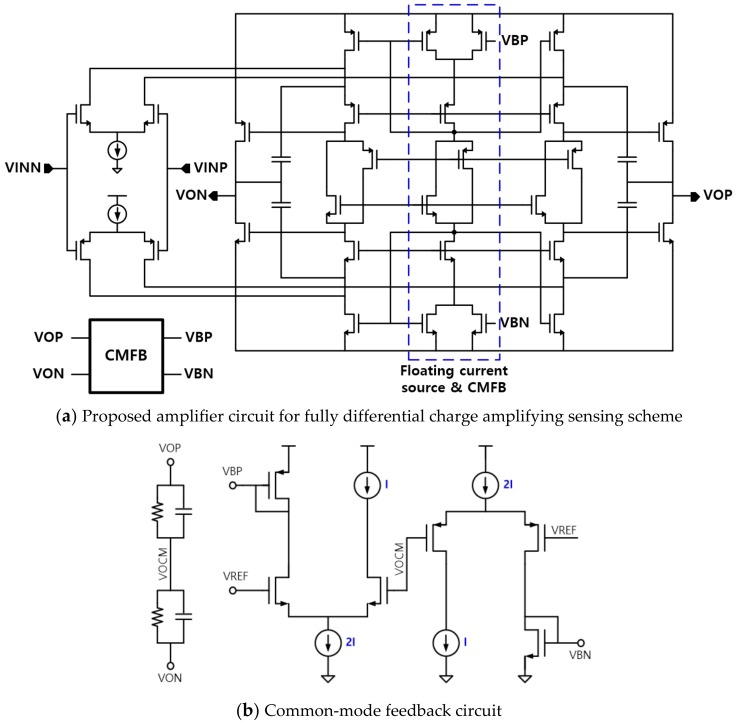
Proposed fully differential amplifier with rail-to-rail input dynamic range and its common-mode feedback circuit.

We designed the fully differential amplifier with an input dynamic range above the supply voltage, as shown in Figure 8a. As complementary pairs of pMOS and nMOS transistors are deployed in the first stage, the input dynamic range of the circuit is extended beyond rail-to-rail. The two output voltages of the amplifier are averaged and fed back to the upper and lower current sources using the common-mode feedback (CMFB) circuit shown in Figure 8b so that the common mode voltage of the output is adjusted to half the power supply. The main performance parameters of the designed amplifier are summarized in Table 1.

### 3.3. Implementation of Other Blocks

**Figure 9:**
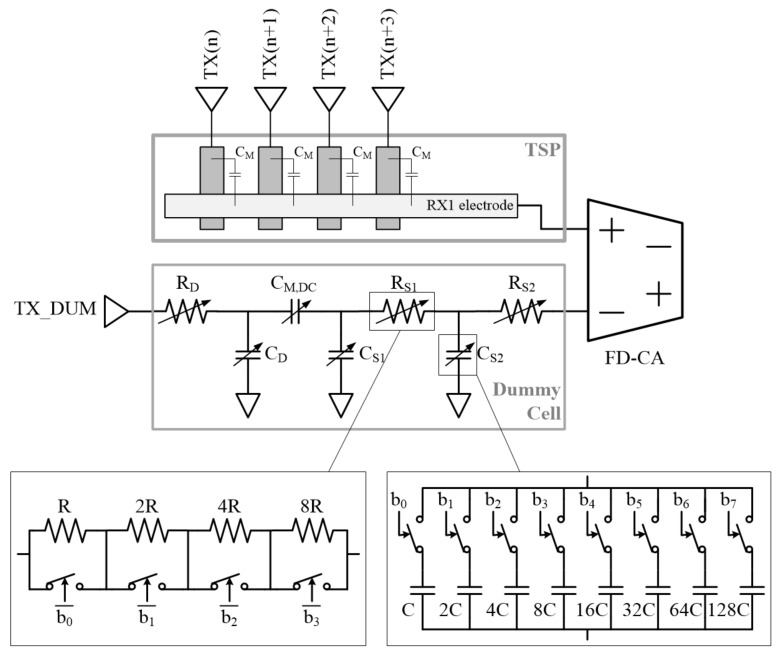
Dummy cell block implemented in proposed TSC and the configuration.

One dummy edge channel is implemented with multiple capacitors and resistors that are variable by register setting, as shown in Figure 9. It pairs with an edge channel and is tuned to have frequency characteristics similar to that of the edge channel. In touch screen sensors using differential sensing without a dummy channel, the number of sensing data acquired in a TX line is one less than the number of RX nodes in the line, so the touch data of each node can be determined as a ratio of a certain reference value. For example, the reference value may be set as the mutual capacitance value of the first RX column (RX1) of the TSP. However, in this case, normal touch data can be obtained only when the mutual capacitance values of all of the nodes in the RX1 column are the same. If a certain RX1 node is touched, a line-shaped offset may occur in the touch data distribution because the reference value of the touched TX row is different than the others. In contrast, since the dummy channel embedded in the chip always provides the same reference value as the no touch condition, line-shaped offset does not occur.

**Figure 10:**
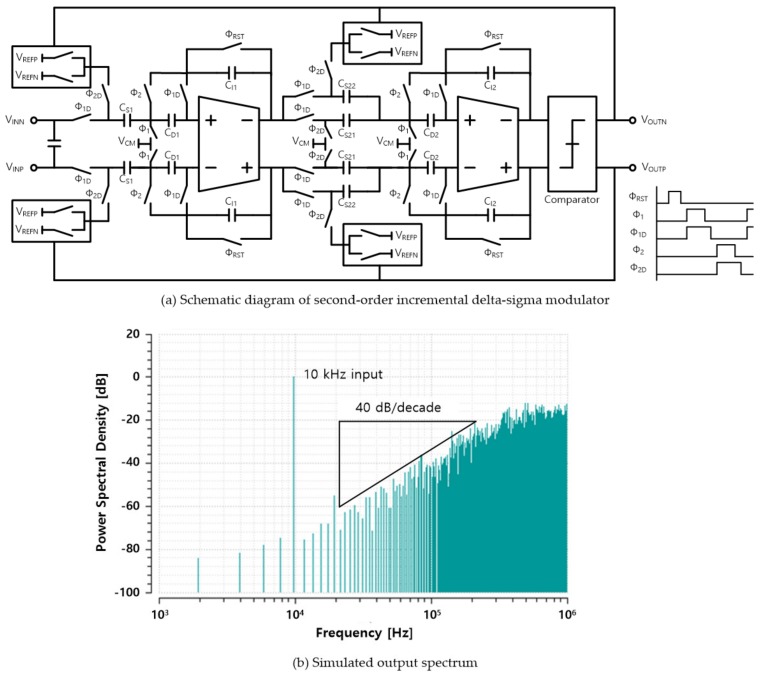
Circuit implementation of second order delta-sigma modulator and the noise-shaping property.

The oversampling ADC is implemented with an incremental delta-sigma ADC that periodically initializes the bit stream, providing an additional enhancement of the SNR by the averaging effect of the signal over one oversampling period [10]. The schematic of delta-sigma modulator is illustrated in Figure 10a. The correlated double sampling (CDS) technique is applied to the two integrators to alleviate the amplifier’s offset and low frequency noises such as flicker noise. Figure 10b shows the second-order noise shaping characteristics of the delta-sigma modulator for a 10-kHz-sinusoidal input.

**Figure 11:**
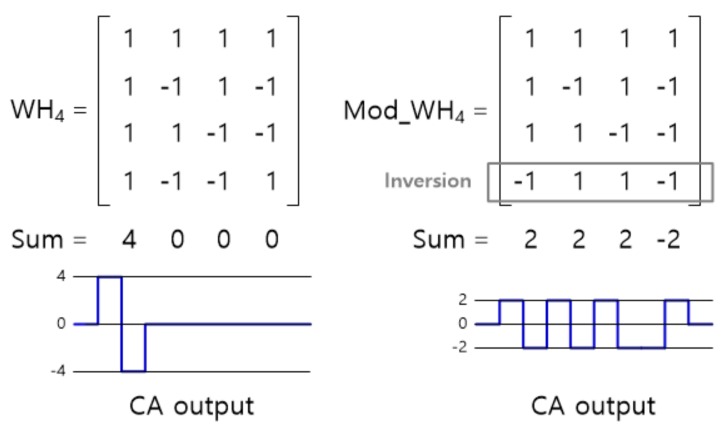
Conventional Walsh–Hadamard (W–H) matrix vs. modified W–H matrix.

To enhance the SNR, the TSC supports a function to simultaneously drive multiple TX channels using orthogonal codes by embedding a table to store the code values [10,11]. As an orthogonal code, the conventional Walsh–Hadamard (W–H) code, which is popular in communication technologies, suffers from the fact that the sum of the first column elements is very large and easily saturates the CA output in TSP sensor applications. To solve this problem, a modified W–H code is used that minimizes the maximum of the sum of each column [20]; Figure 11 shows an example of the modified code. 

On the other hand, although the differential sensing method shows excellent immunity to display noise, it can be vulnerable to external noise coming in different phases and magnitudes into adjacent channels. This work employs three techniques to reduce the external noise. First, the touch sensor drives the sensing signal at the frequency with the lowest noise component through a frequency analysis of the noise. The frequency spectrums of major noise sources such as chargers and lamps are already well known by previous studies [10,12] and the TX driver was implemented to select the optimum frequency of the driving signal. Second, the 2nd-order BPF in the AFE block significantly attenuates the out-of-band noise different from the driving frequency. Third, the external noise is further reduced by temporally averaging several frames of touch data in the digital block.

A low-drop output (LDO) regulator was implemented to prevent power-supply noise from degrading touch performance. The measured PSRR of the circuit exhibits 72 dB at 60 Hz (HUM noise) and 70 dB at 240 kHz (signal frequency for touch sensing).

## 4. Results and Discussion 

**Figure 12:**
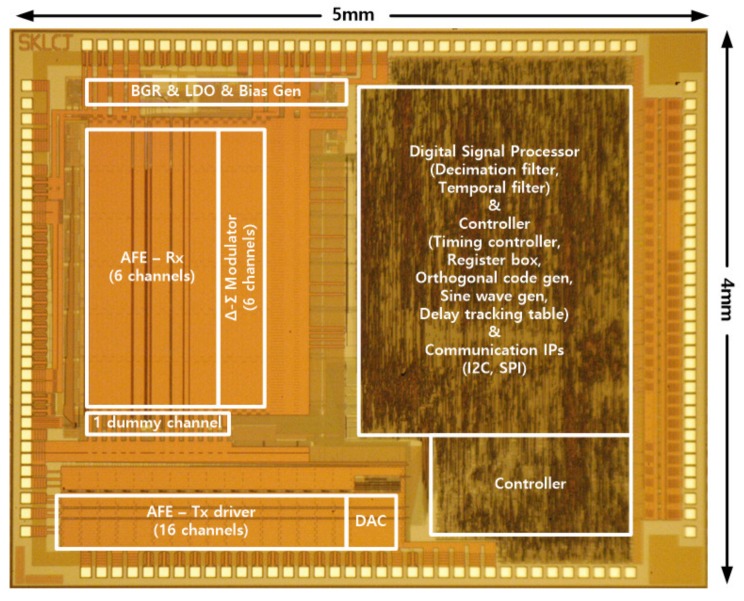
Photomicrograph of the fabricated TSC.

Figure 12 is a microphotograph of the test chip fabricated using a 0.35-μm 1P4M process. The test chip was composed of TX drivers that can drive 15 TX sensor electrodes and six columns of AFEs and delta-sigma ADCs to process 12 RX sensor electrodes in a TSP. In the experiment, the TSP with 15-TX and 10-RX sensor electrodes was used. Decimation filters for the delta-sigma ADC, registers to control the TSC and a timing controller were also designed in the digital block of the test chip. 

**Figure 13:**
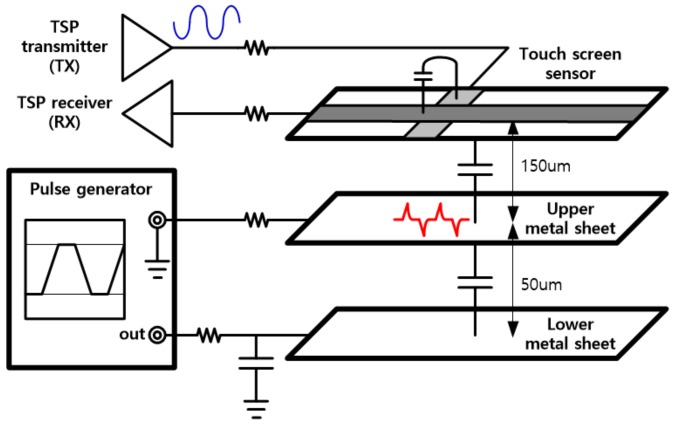
Experimental environment for inserting display noise into the TSP.

To mimic a display module with large coupling capacitance, two thin metal sheets were stacked beneath the TSP and electrically isolated. Here, the distance from the RX electrode to the upper sheet is approximately 150 um. The upper metal sheet was connected to ground through a small resistor to operate like a display common electrode. A pulse that emulates display driving signals was inserted into the lower metal sheet through an RC low-pass filter to generate display noise. By properly selecting the values of the resistor and the capacitor in the filter, the slope of the pulse was adjusted to be similar to the actual waveform. Figure 13 depicts the experimental environment for evaluation.

**Figure 14:**
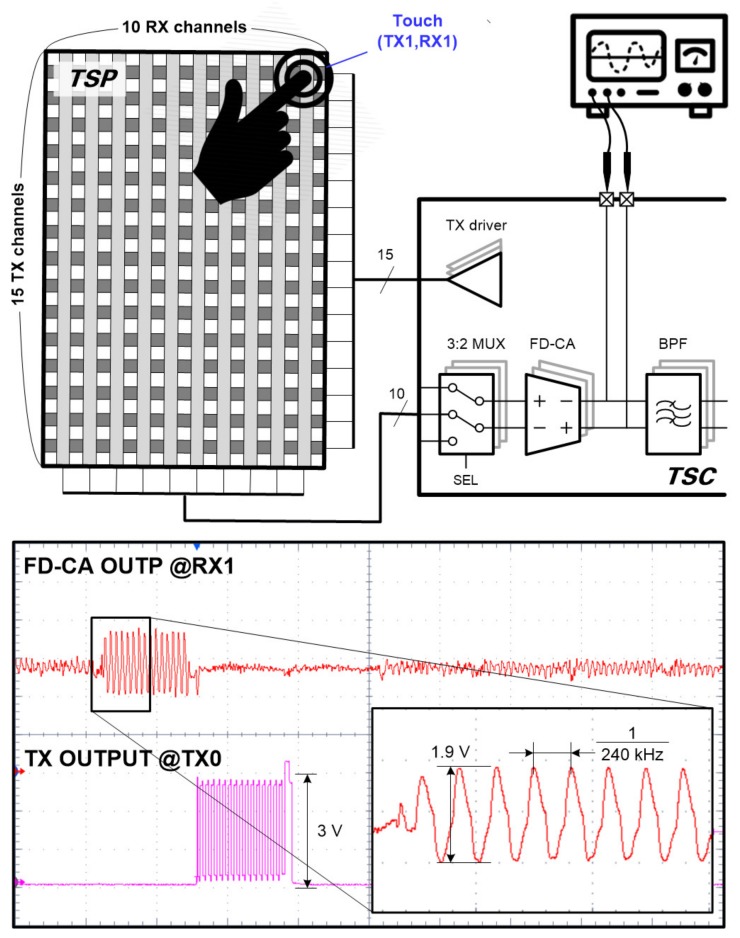
Measured output of FD-CA at the touch position.

**Figure 15:**
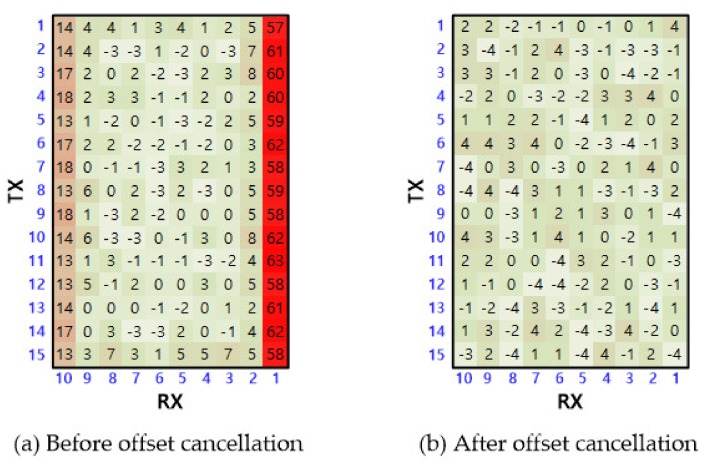
Touchscreen data of each node before and after offset cancellation measured in on touch state.

Figure 14 shows the waveform measured at the FD-CA output when the (TX1, RX1) sensor node was touched. Here, the output of TX0 was used as a trigger signal to ensure that the measuring probes did not affect touch operation. When a 6-mm-diameter metal pillar touched the screen, the FD-CA output had a larger voltage swing than that of the other nodes. On the other hand, waveforms with small amplitudes appeared at locations where no touch occurred, which may have been caused by a mismatch of mutual capacitances between RX channels. The touch data offsets resulting from the mismatch were eliminated by subtracting the touch data measured during operation time from the premeasured data in the no touch state. The touchscreen data in Figure 15 demonstrates that the offset cancellation technique is effective to mitigate the mismatch.

**Figure 16:**
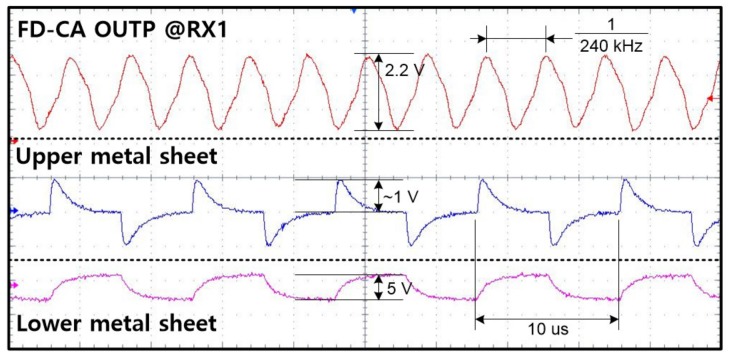
Noise immunity of FD-CA circuit against display noise.

The immunity of the proposed FD-CA against display noise is shown in Figure 16. A driving signal for display used a square wave with a 100-kHz frequency, 1-us rising or falling time, and 5-V swing. The resistance connected to the upper sheet was adjusted so that the excited voltage of the common electrode by display stimulus returns to the reference voltage within one writing period. The waveform of the first row in Figure 16 demonstrates that even strong display noise with a peak of about 1 V has little effect on the output of the FD-CA.

**Figure 17:**
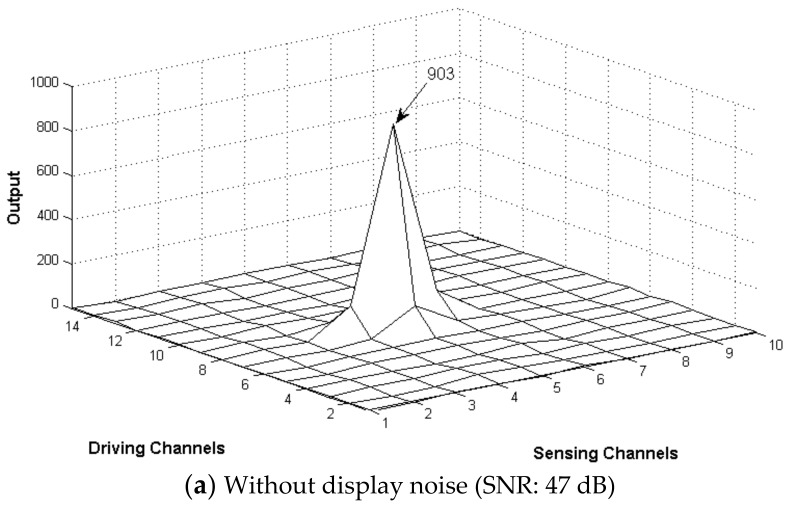

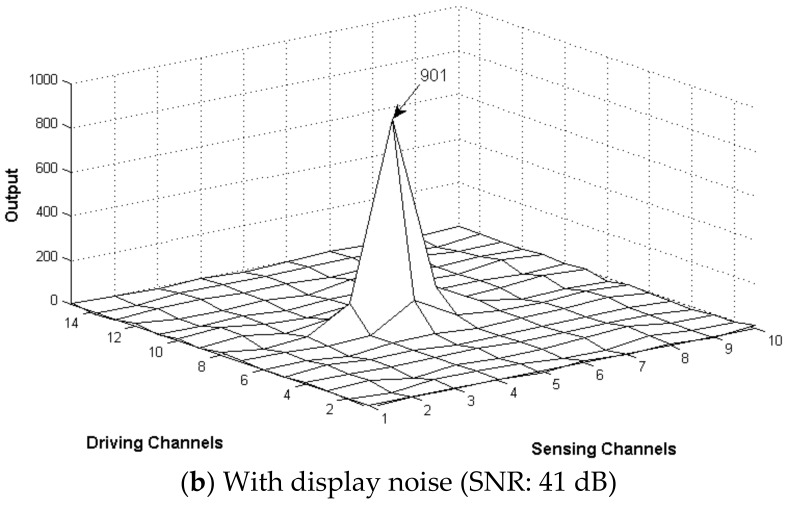
Distribution of touch data with and without display noise when a 6-mm diameter metal pillar touches the screen.

To evaluate the operation of the two-dimensional touch sensor, the TX driver generated square waves with a voltage swing of 3.3 V at 240 kHz and drove four channels simultaneously. In addition, 30 pulses were assigned to one chip of the orthogonal code, thereby driving at a frame refresh rate of 250 Hz. Figure 17 shows distribution profiles of the touch data with and without display noise when a 6-mm-diameter metal pillar touched the center of the TSP, resulting in SNRs of 41 dB and 47 dB, respectively. Compared to the case without display noise, the fluctuation of the touch values slightly increased when the noise was applied, resulting in a decrease of the SNR. Nevertheless, if touch coordinates are calculated by applying the weighted average to the touch data in the 3 x 3 nodes around a touch, the SNR of 41 dB means that the maximum accuracy error is about 0.4 mm on a TSP with a 6-mm sensor pitch. However, in most cases, the accuracy error is much smaller than the maximum value because the maximum error can occur only when the noise of 3σ is symmetrical with an opposite sign in the diagonal direction of the touch position, which rarely occurs. Therefore, the above SNR performance confirms that the test chip has excellent noise immunity even in a strong display noise environment.

Table 2 summarizes the performance of the test chip and compares it with the previous remarkable readout ICs for touch sensing. Compared to the study [11] using the most similar structure, the proposed test chip showed a slightly smaller SNR in the environment without external noise. However, when the noise was strong, the test chip achieved a higher SNR than the other previous work. On the other hand, a recent study [21] shows outstanding performance in terms of SNR. Unfortunately, the accurate comparison between the two works is difficult because the evaluation conditions such as display noise pattern are not clearly presented in [21].

In summary, a quantitative analysis of the characteristics of display noise in the time domain is presented in this paper. Based on the analysis, it is demonstrated that the proposed fully differential amplifier with an input dynamic range wider than the maximum display noise peak can effectively suppress display noise with low power consumption and compact size, even in thin displays. The proposed circuit approach to display noise has potential as a useful solution in emerging displays such as flexible OLED displays.

## Figures and Tables

**Table 1:** Main performance of a fully differential amplifier with wide input dynamic range.

Parameter	Value	Unit
Supply voltage	3	V
Input common-mode range	−0.3–3.3	V
CMRR at 240 kHz		
V_common_:		
from −0.3 V to 0.9 V	72	dB
from 1.1 V to 1.9 V	77	dB
from 2.1 V to 3.3 V	71	dB
Open-loop gain (NN, 27 °C)	85	dB
Unity-gain frequency	5	MHz
Unity-gain phase margin	63	°
DC current	28	uA
Peak current	480	uA
Slew rate	9.4	V/us
Settling time (1%), Vstep = 1 V, C_L_ = 2 pF	212	ns
Unity-gain frequency of CMFB circuit (R: 200 kΩ, C: 0.1 pF)	6.1	MHz

**Table 2:** Performance comparison.

		ISCAS 19 [21]	ISSCC 16 [22]	IEEE Sensors 16 [23]	T-CAS 16 [11]	This Study
Process		0.13 μm	0.18 μm	0.35 μm	0.18 μm	0.35 μm
Supply voltage		N/A	2.7~3.3 V	3.3 V	3.3 V	3.3 V
Channel		TX: 31 RX: 15	TX: 36 RX: 64	TX: 12 RX: 16	TX: 32 RX: 10	TX: 15 RX: 12
TX diving multiplexing		CDM	CDM	TDM	CDM	CDM
Charge amplifier		Single-ended	Single-ended	Fully differential	Fully differential	Fully differential
SNR	Display Off	N/A	54 dB	35.5 dB	72 dB	47.1 dB
	Display On	54.2 dB ^(1)^	N/A	27.5 dB	36.1 dB	41.0 dB ^(2)^
Frame rate		240 Hz	120 Hz	175 Hz	240 Hz	250 Hz
Power		11.5 mW	94.5 mW (including digital block)	76 mW	TX: 34.2 mW RX: 8.43 mW	10.4 mW
Area		1.49 mm^2^	36 mm^2^	5.02 mm^2^	1.25 mm^2^	4.1 mm^2^
TSP type		Mutual	Mutual	Mutual	Mutual	Mutual

^(1)^ Display noise pattern: not clearly presented. ^(2)^ Display noise pattern: sharp triangular-wave-shaped pulses with 1-V peak at 100 kHz.
